# Supplementary material for: Association between use of antihypertensives and cognitive decline in the elderly—A retrospective observational study
Source: PLoS One. 2023 Dec 20;18(12):e0295658. doi: 10.1371/journal.pone.0295658 (PMC10732389; doi:10.1371/journal.pone.0295658)
Supplement: Attachment — Submitted filename: Oct. 31 a Response to Reviewers - Version 2.docx [file pone.0295658.s001.docx]

Dear Editor,

Thank you to the editor and reviewers for their suggestions. We have addressed all the suggestions and comments as described below, and all the changes in the manuscript are high lightened:

Journal Requirements:

When submitting your revision, we need you to address these additional requirements.

1. Please ensure that your manuscript meets PLOS ONE's style requirements, including those for file naming. The PLOS ONE style templates can be found at 
<https://journals.plos.org/plosone/s/file?id=wjVg/PLOSOne_formatting_sample_main_body.pdf> and 
<https://journals.plos.org/plosone/s/file?id=ba62/PLOSOne_formatting_sample_title_authors_affiliations.pdf>

Authors: We have made sure that the PLOS ONE’s style requirements have been incorporated in the manuscript.

2. We noticed you have some minor occurrence of overlapping text with the following previous publication(s) among others, which needs to be addressed:

(a) ALAGIAKRISHNAN, K., DHAMI, P., SENTHILSELVAN, A.. Predictors of Conversion to Dementia in Patients With Mild Cognitive Impairment: The Role of Low Body Temperature. Journal of Clinical Medicine Research, North America, 15, apr. 2023. Available at: <<https://www.jocmr.org/index.php/JOCMR/article/view/4883/25893713>>. Date accessed: 14 Sep. 2023.

(b) Cuevas C, Ryan N, Quirós A, Del Angel JG, Gonzalo N, Salinas P, Jiménez-Quevedo P, Nombela-Franco L, Nuñez-Gil I, Fernandez-Ortiz A, Macaya C, Escaned J. Determinants of percutaneous coronary intervention success in repeat chronic total occlusion procedures following an initial failed attempt. World J Cardiol 2017; 9(4): 355-362

(c) Cuevas C, Ryan N, Quirós A, Del Angel JG, Gonzalo N, Salinas P, Jiménez-Quevedo P, Nombela-Franco L, Nuñez-Gil I, Fernandez-Ortiz A, Macaya C, Escaned J. Determinants of percutaneous coronary intervention success in repeat chronic total occlusion procedures following an initial failed attempt. World J Cardiol 2017; 9(4): 355-362 [PMID: 28515854 DOI: 10.4330/wjc.v9.i4.355]

In your revision ensure you cite all your sources (including your own works), and quote or rephrase any duplicated text outside the methods section. Further consideration is dependent on these concerns being addressed.

Authors:

There is some overlap only in the methods section with the first article (Alagiakrishnan et al.). Our article does not have any overlap with article by Cuevas et al.

3. Please note that funding information should not appear in any section or other areas of your manuscript. We will only publish funding information present in the Funding Statement section of the online submission form. Please remove any funding-related text from the manuscript.

Authors:

We have removed funding information from the manuscript and included in the online submission form.

4. We note that the grant information you provided in the ‘Funding Information’ and ‘Financial Disclosure’ sections do not match.

When you resubmit, please ensure that you provide the correct grant numbers for the awards you received for your study in the ‘Funding Information’ section.

Authors:

Financial disclosure has been removed from the manuscript. Funding information and competing interest section have been updated in the cover letter.

5. In your Data Availability statement, you have not specified where the minimal data set underlying the results described in your manuscript can be found. PLOS defines a study's minimal data set as the underlying data used to reach the conclusions drawn in the manuscript and any additional data required to replicate the reported study findings in their entirety. All PLOS journals require that the minimal data set be made fully available. For more information about our data policy, please see <http://journals.plos.org/plosone/s/data-availability>.

"Upon re-submitting your revised manuscript, please upload your study’s minimal underlying data set as either Supporting Information files or to a stable, public repository and include the relevant URLs, DOIs, or accession numbers within your revised cover letter. For a list of acceptable repositories, please see <http://journals.plos.org/plosone/s/data-availability#loc-recommended-repositories>. Any potentially identifying patient information must be fully anonymized.

Important: If there are ethical or legal restrictions to sharing your data publicly, please explain these restrictions in detail. Please see our guidelines for more information on what we consider unacceptable restrictions to publicly sharing data: <http://journals.plos.org/plosone/s/data-availability#loc-unacceptable-data-access-restrictions>. Note that it is not acceptable for the authors to be the sole named individuals responsible for ensuring data access.

We will update your Data Availability statement to reflect the information you provide in your cover letter.

Authors

According to restrictions placed on us by Alberta health services, we are not permitted to share any data regarding the patients in this study. The data we are allowed to make available is presented in the tables and manuscript.

Data Availability statement: “The authors confirm that the data supporting the findings of this study are available within the article.”

6. We note you have included a table to which you do not refer in the text of your manuscript. Please ensure that you refer to Table 2 in your text; if accepted, production will need this reference to link the reader to the Table.

Authors:

We have now referred Table 2 in line 136 page 8 of the text.

7. Please include captions for your Supporting Information files at the end of your manuscript, and update any in-text citations to match accordingly. Please see our Supporting Information guidelines for more information: <http://journals.plos.org/plosone/s/supporting-information>.

Authors:

We do not have any supporting information files in the manuscript that require citations.

Additional Editor Comments:

Two reviewers have an interest in this research; however, major concerns are raised in the present study. See the suggestions carefully, and respond to them appropriately.

[Note: HTML markup is below. Please do not edit.]

Reviewers' comments:

Reviewer's Responses to Questions

**Comments to the Author**

1. Is the manuscript technically sound, and do the data support the conclusions?

The manuscript must describe a technically sound piece of scientific research with data that supports the conclusions. Experiments must have been conducted rigorously, with appropriate controls, replication, and sample sizes. The conclusions must be drawn appropriately based on the data presented.

Reviewer #1: Yes

Reviewer #2: No

2. Has the statistical analysis been performed appropriately and rigorously?

Reviewer #1: Yes

Reviewer #2: No

3. Have the authors made all data underlying the findings in their manuscript fully available?

The [PLOS Data policy](http://www.plosone.org/static/policies.action#sharing) requires authors to make all data underlying the findings described in their manuscript fully available without restriction, with rare exception (please refer to the Data Availability Statement in the manuscript PDF file). The data should be provided as part of the manuscript or its supporting information, or deposited to a public repository. For example, in addition to summary statistics, the data points behind means, medians and variance measures should be available. If there are restrictions on publicly sharing data—e.g. participant privacy or use of data from a third party—those must be specified.

Reviewer #1: Yes

Reviewer #2: Yes

4. Is the manuscript presented in an intelligible fashion and written in standard English?

PLOS ONE does not copyedit accepted manuscripts, so the language in submitted articles must be clear, correct, and unambiguous. Any typographical or grammatical errors should be corrected at revision, so please note any specific errors here.

Reviewer #1: Yes

Reviewer #2: No

5. Review Comments to the Author

Please use the space provided to explain your answers to the questions above. You may also include additional comments for the author, including concerns about dual publication, research ethics, or publication ethics. (Please upload your review as an

Reviewer #1: The authors examined the factors that contribute to the conversion from MCI to dementia and showed that the beta blocker is protective.
 Ihave several major comments for this paper.
In line 68, please spell out CCBs.

Authors:

We have now spelled out CCBs in line 65 in page 4 of the manuscript.

In line 74, I think that 143 patients develop dementia after 5.5 years of observation of 335 MCI patients is too high a frequency. Please elaborate on the inclusion criteria for 335 patients.

Authors:

We have now elaborated the inclusion/exclusion criteria in the methods section in lines 76 to 784, page 4 of our manuscript.

In line 81, if the data for patients seen in 2021 were compiled in 2021, wouldn't it include patients with little or no observation period? The longer the observation period, the higher the risk of CONVERTER, so the observation period should be included in the COVARIATE.

Authors:

Patient charts from geriatric patient assessments conducted from 2015 to 2018 were retrieved for the study. The baseline of the study was between 2015 and 2018 and the patients were then followed up for at least three years with an average of 5 years. None of the patients seen for the first time in 2021 was included in the study.

In line 86, Please be specific about the diagnostic criteria, not just cite them, as the diagnosis of dementia and MCI is important.

Authors:

We have now included diagnostic criteria of MCI and dementia in the methods section, line 87 to 97, page 5 of our manuscript.

In line 109 and Table 1, please describe what type of dementia the subject has developed, is it AD or DLB? Is it also vascular dementia?

Authors:

The types of dementia are now included in line 115-119, page 6, of the manuscript.

In line 115, there was significant differences in age, sex, family history of dementia, MMSE scores, MoCA scores, SBP, and diastolic blood pressure (DBP) between patients who converted from MCI to dementia relative to non-converters. I think more attention should be paid to the higher blood pressure in the Converter group. Does Multiple logistic analysis include blood pressure?

Authors:

We initially included systolic and diastolic blood pressure in the logistic regression analysis. As they were no longer significant after accounting for other variables, they were removed from the final model.

In line 127, authors discussed that BB usage was significantly protective against conversion, as patients with MCI taking BBs at baseline were less likely to develop dementia in the follow up period. I think this is an oversimplification. Could it be considered that patients being treated with BB have a reduced risk of dementia due to better blood pressure control? It would be good to compare blood pressure for each patient on each drug to clarify this.

Authors:

In our study, systolic and diastolic blood pressure was included in the logistic regression, and they did not confound the association between use of beta-blocker and conversion from MCI to dementia. This indicates it is unlikely that better blood pressure control is why beta-blocker use reduced risk of dementia.

In line 146, a preclinical study showed that beta 2-adrenergic activation enhances neurogenesis (21). If this is true, wouldn't BB decrease neurogenesis and put us at risk for dementia?

Authors:

Thank you for pointing out this information. We have clarified these points in the discussion section on line 166-175 page 10 of the manuscript. Although this one preclinical study points out that use of beta-blockers is associated with decreased neurogenesis and possible worsening of cognitive outcomes, a post-mortem study and a recent human study points out the possibility of a protective effect, which is also seen in our study. We have added these references to the manuscript as well.

In Discussion, please discuss the importance of blood pressure control as blood pressure is significantly higher in CONVERTER. Recently, it has been reported that hypertension may increase the risk of AD and other dementias by causing stagnation of the glymphatic pathway due to enlargement of the perivascular space (e.g. PMID: 37537887).

Authors:

We have now elaborated on the importance of BP control, and included an additional reference in line 186-187 in page 10,11 of our manuscript.

Reviewer #2: Thank you for allowing me to review the manuscript entitled “Association between use of antihypertensives and cognitive decline in the elderly -A retrospective observational　study.” This retrospective observational study revealed that the patients who were on beta-blockers (BBs) had a 57% reduction in the odds of converting to dementia. According to my opinion this study is unique, novel, and is of great interest. However, before this manuscript can be considered suitable for publication, several major concerns must be addressed.

1) Please revise the table to make it easier for readers to understand. What do the numbers in the table represent? I would like you to check the number and p-value in Current or past smokers because I think that they may be wrong.

Authors

We have revised Table 1 to make it easier for the readers to understand. We thank the reviewer for alerting us to the incorrect frequencies and p-values for current and past smokers. We have now corrected the error. This change can be found on page 7 of the manuscript.

2) I think that MCI is reversible. Therefore, the patients who have reverted to healthy individuals should be excluded. In addition, the patients whose type of antihypertensive drug has changed may be excluded too. The authors can identify them.

Authors:

In our study, none of the patients reverted to normal cognitive function for age. Patients were diagnosed at the Seniors Clinic based on subjective/objective criteria (as described in text) by trained geriatricians familiar with MCI diagnosis.

3) Did the authors include the all dementia? Why did not they just focus on AD?

Authors:

We included all types of dementia in line 115-119 on page 6 of the manuscript. The sample size was not adequate to focus on one type of dementia.

4) I think it would be better to clearly state the hypothesis of this research in Background section.

Authors:

We have now added the hypothesis information at the end of the introduction section of the article in line 72-73 on page 4.

5) There are differences in the backgrounds of the two groups. In my opinion, propensity score matching may be appropriate in this study. Please reconsider the statistical analysis.

Authors:

The sample size is too small to conduct propensity score analysis. There were only 69 beta-blockers users, and this sample size is too small to consider beta-blockers as the main exposure in the propensity score analysis.
