# Supplementary material for: Association between use of antihypertensives and cognitive decline in the elderly—A retrospective observational study
Source: PLoS One. 2023 Dec 20;18(12):e0295658. doi: 10.1371/journal.pone.0295658 (PMC10732389; doi:10.1371/journal.pone.0295658)
Supplement: Attachment — Submitted filename: Nov. 21- Response to reviewers comments.docx [file pone.0295658.s002.docx]

6. Review Comments to the Author

Reviewer #2: Thank you for allowing me to review the revised manuscript entitled “Association between use of antihypertensives and cognitive decline in the elderly -A retrospective observational study.” I think that the authors generally responded to comments. In this study, the small sample size might cause the statistical analysis problem, which may change the interpretation of the results. Although the authors mention the sample size in the limitation section, it's not enough and I would like you to discuss fully.

Authors:

We thank the reviewer for the positive comment about our response to the reviewer’s previous comments. We have now included the following statement in the limitation section on the statistical power of our study with a sample size of 335 subjects.

*“In this study, the total sample size of 335 subjects with 191 in the non-converter group and 144 in the converter group had an adequate statistical power of 80% or greater with 5% level of significance to detect a difference of 12% or greater in the baseline characteristics between the two groups. Due to the small number of users of specific medications within each antihypertensive drug class in this study, we could not examine the effect of specific medications on conversion from MCI to dementia.”*
